# Airborne *Alternaria* Spores: 70 Annual Records in Northwestern Spain

**DOI:** 10.3390/jof10100681

**Published:** 2024-09-29

**Authors:** Kenia C. Sánchez Espinosa, María Jesús Aira, María Fernández-González, Francisco Javier Rodríguez-Rajo

**Affiliations:** 1 Department of Plant Biology and Soil Sciences, Faculty of Sciences, University of Vigo, 32004 Ourense, Spain; mfgonzalez@uvigo.gal (M.F.-G.); javirajo@uvigo.gal (F.J.R.-R.); 2 Department of Botany, Faculty of Biology, University of Santiago de Compostela, C/ Lope Gómez de Marzoa, s/n., 15782 Santiago de Compostela, Spain; mariajesus.aira@usc.es

**Keywords:** airborne spores, *Alternaria*, NW Iberian Peninsula, meteorological parameter, allergy

## Abstract

This study presents data on the concentration of *Alternaria* spores in the atmosphere of the northwestern Iberian Peninsula. A non-viable volumetric method was used to collect the samples, resulting in a database of 70 annual. When the annual averages for each locality are calculated, Ourense stands out with 2152 spores/m^3^, followed by Vigo and Lugo, while Santiago de Compostela recorded the lowest concentrations. Focusing on the total data for each locality, the main sporulation period started in May and ended in October in all localities, centered on a single phase, with an earlier onset and longer duration in Ourense and Vigo. The number of days with concentrations in excess of 100 spores/m^3^ was very low in Santiago de Compostela, Ourense, and Vigo and null in Lugo. Temperature was the meteorological parameter for which the highest statistical correlation was obtained in all locations, being favorable to the concentration of spores in the air. Temperature ranges favorable to the presence of airborne spores in the study area ranged from 25.5 to 31.2 °C. Based on the analysis of the data collected, it is concluded that *Alternaria* spore concentrations are low throughout most of the year.

## 1. Introduction

Airborne fungal spores represent a significant part of the bioaerosol, and the most common worldwide are *Cladosporium* and *Alternaria*, a reflection of the cosmopolitan character of the numerous species included in both genera [1,2,3]. *Alternaria* Nees belongs to the family Pleosporaceae, order Pleosporales, class Dothideomycetes, phylum Ascomycota [4]. It is a ubiquitous fungal genus that encompasses 26 sections and 360 species [5], many of them saprobic, endophytic, and pathogenic.

The identification of fungal spores and their quantification in the atmosphere has various applications, and numerous studies have investigated this phenomenon in various parts of the world [6,7,8]. From a clinical point of view, the involvement of fungi in various respiratory pathologies, such as rhinitis and allergic asthma, has been known since ancient times. Given their opportunistic nature, many of them can cause systemic and dermatological infections in immunocompromised patients and transplant recipients [9,10,11]. 

*Alternaria* is one of the genera most frequently cited as a cause of allergic respiratory diseases, especially in children [12]. Its ability to induce these reactions is due to the presence of water-soluble proteins that are extracted by the mucous fluids of the respiratory tract. Among its most clinically significant allergens are Alt a 1, Alt a 4, Alt a 8, and Alt a 13 [13,14,15]. At present, it is difficult to estimate the real prevalence of *Alternaria* sensitization since there has been a lack of standardization in the way samples are extracted for diagnosis [16], and it may be underestimated as a consequence.

In addition, various species of *Alternaria* have been isolated from soil samples and a wide range of plant species, on which they can act as important phytopathogens. Recently, Schmey et al. [17] reported that several species from the sections Porri, Alternaria, and Ulocladiodes cause foliar diseases in potato and tomato crops, known as early blight or leaf sporulation. Among the main effects they have on these crops are plant defoliation, fruit necrosis, and rotting, as well as a reduction in crop yield. *Alternaria* has also been reported as a causal agent of diseases in apple, cherry, pear, and maize crops [18]. Moreover, *Alternaria* causes several diseases in citrus species, with the consequence that in several countries screening measures are already being implemented to locate areas that are climatically more suitable for such crops [19]. It has been estimated that the presence of fungal spores in the air can produce economic losses of 30–50% in fruit and vegetable storage facilities [20], with *Alternaria alternata* being frequently present in apple and potato stores [18,21,22]. Some species of *Alternaria* synthesize more than 70 types of mycotoxins, which can represent a danger to consumers and producers [23,24], among them AF-toxin I, II, and III, detected in strawberry crops, and TA1, TA2, TB1, TB2, TC1, TC2, TD1, TD2, TE1, and TE2, identified in tomato crops. These toxins mainly exhibit phytotoxic, cytotoxic, and antimicrobial properties [25].

A less studied aspect of the negative effects of *Alternaria* is its role as a biodeteriorating agent. The extracellular enzymes and acid metabolites it produces have aesthetic repercussions and can compromise the integrity of various materials [26,27]. Several species of this genus have been isolated from oil and mural paintings, wooden artworks, audiovisual materials, and textiles [28,29]. 

The identification and enumeration of *Alternaria* spores in the air is an important tool for monitoring human health, air quality, and the biodiversity of the environment and can provide valuable information for decision-making in the areas of public health and environmental and heritage conservation. 

The presence of *Alternaria* spores in the atmosphere is frequently reported in a range of geographical regions [3,30,31,32]. The production and release of its spores result from a complex interplay of several factors that influence mycelial growth and development, as well as sporulation. These include temperature, humidity, water, and nutrient availability [33,34,35]. On the other hand, once in the air, fungal spores are subject to atmospheric dynamics influenced by factors such as the climatic pattern of each area, topography, the geographical barriers of the territory, and human activity. Therefore, both the concentration and the seasonal pattern of spores vary widely depending on the location and geographical characteristics of the sampling station. At present, aeromycological studies of *Alternaria* that include extensive time series data are limited. The main goals of the present study were therefore twofold: (1) to determine the spatial and temporal variations in the atmospheric concentrations of *Alternaria* spores in the northwestern quadrant of the Iberian Peninsula; and (2) to analyze the influence of temperature, relative humidity, and precipitation on the production and dispersion of these spores.

## 2. Materials and Methods

### 2.1. Characteristics and Location of the Study Area

**Figure 1:**
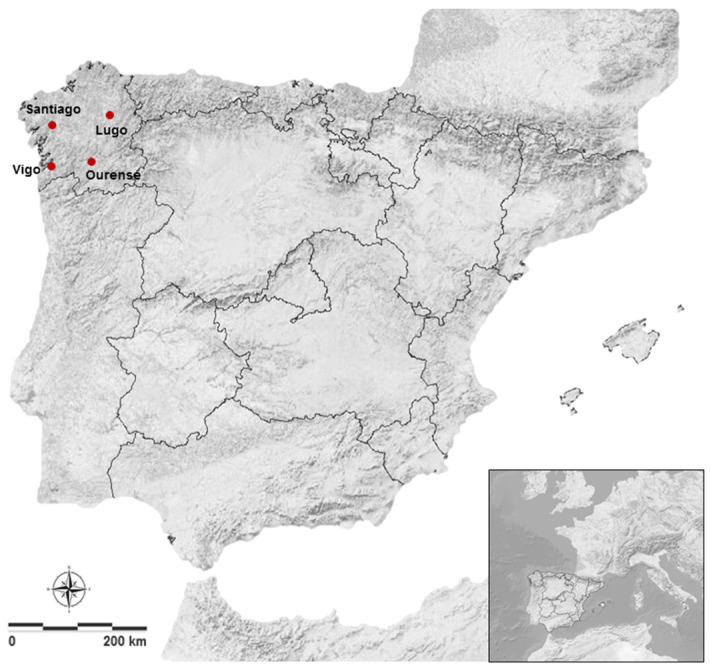
Location of the aerobiological stations included in the study on the Iberian Peninsula.

The aerobiological data used in this study is derived from stations located in the urban environment of four cities in the northwestern part of the Iberian Peninsula (Figure 1). Santiago de Compostela (referred to simply as Santiago in the tables and figures) is located at an altitude of 270 m.a.s.l (42°53′ N, 8°32′ W), Ourense at 138 m.a.s.l (42°21′ N, 7°51′ W), Vigo at 50 m.a.s.l (42°14′ N, 8°43′ W), and Lugo at 452 m.a.s.l (43°00′ N, 7°33′ W).

In phytogeographical terms, this territory belongs to the Atlantic-European Subregion of the Eurosiberian Region and has a temperate macro-bioclimate. The coastal areas have a more temperate climate than those of the interior zone, where the annual thermal amplitude is greater [36,37]. 

### 2.2. Sample Collection, Identification and Spore Counting

Sampling was conducted between 1993 and 2023. Table 1 provides information about the database used in this study, which draws on a total of 70 annual records. The city with the most years of sampling was Santiago de Compostela (20 years), followed by Ourense (19 years), Vigo (18 years), and Lugo (13 years). The years of sampling in each locality were not previously determined but are a reflection of logistical limitations, time restrictions in the financing of the research project, and difficulties in accessing the stations due to the COVID-19 pandemic. Although the records are neither continuous nor uniform across all locations, the methodology proposed by the Spanish Aerobiology Network [38] was used in all samplings to ensure the reliability and homogeneity of the data. The study was resumed in 2022 and 2023 at the four sites, with the aim of analyzing possible variations in comparison with previous years and analyzing all four stations in a recent period.

The authors collected daily spore concentrations using a Hirst-type 7-day volumetric trap, made either by Burkard Manufacturing Co. Ltd., Rickmansworth, UK, or Lanzoni S.R.L., Bologna, Italy, both operating at a rate of 10 liters per minute. Spores were captured on Melinex tape, which was then divided into daily segments. The average daily concentration of the number of fungal spores was measured using an optical microscope with magnifications of ×400 and ×1000 along two complete longitudinal traverses. Conidia corresponding to the morphological characteristics described for *Alternaria* by Woudenberg et al. [39] were quantified. These were ovoid, obovoid, cylindrical, narrowly ellipsoid or obclavate conidia, with or without beaks, pale olive-brown or medium-brown, smooth or verrucose, with transverse septa, and with or without oblique or longitudinal septa. The septa could be thick, dark, and rigid and form an internal cell-like structure. Spore counting was performed at the generic level since the use of a non-viable method does not allow specific differentiation. To delimit the period with the highest concentration of spores, the authors followed the method proposed by Nilsson and Persson [40]. Thus the Main Spore Season–Global (MSS–G) was calculated, applying this criterion to the average of the daily data in the years of each location, as was the Main Spore Season–Annual (MSS–A), with the daily data of each of the years analyzed.

### 2.3. Meteorological Data and Statistical Analysis

The values of precipitation, humidity, and temperature (maximum, minimum, and mean) were obtained from the records of the Galician Institute for Meteorology and Oceanography, METEOGALICIA [41], using the stations closest to the sampling points.

To establish the relationship between spore counts in the air and meteorological variables, the Spearman rank correlation coefficient was determined (*p* < 0.05), and the correlation was plotted with the corrplot package. The statistical software package used was RStudio Desktop 2024.04.0 + 735.

In addition, a principal component analysis (PCA) was performed to evaluate the overall meteorological influence of all variables on *Alternaria* concentrations in each city. To determine the average temperature that favored the highest presence of these spores in the air, LOESS regressions were performed for each sampling station, taking into account daily values. The purpose of this regression was to identify general patterns in the relationship between both variables, with the value of α = 0.75 [42]. 

## 3. Results

### 3.1. Global Analysis of Spore Concentration

**Figure 2:**
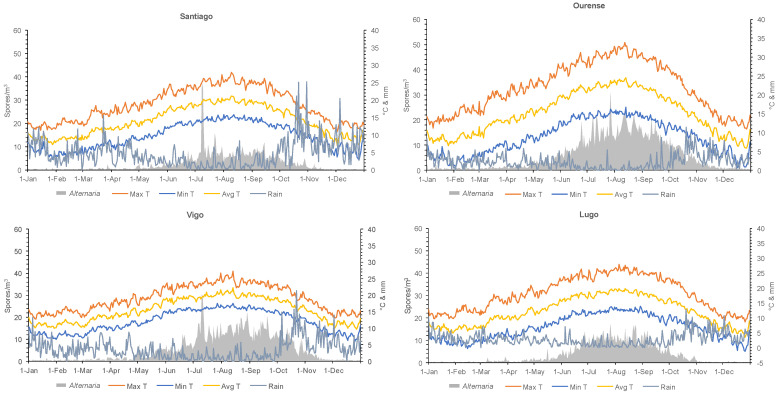
Mean daily concentrations of *Alternaria*, average temperature (maximum temperature—Max T, minimum—Min T, and average—Avg T), and rainfall during the study period in Santiago de Compostela, Ourense, Vigo, and Lugo.

To analyze the data obtained at the four aerobiological stations, the number of years available at each station, the total values, and the annual and daily averages of *Alternaria* concentrations, as well as the meteorological data, were all taken into account (Table 1, Figure 2).

The highest number of spores, in terms of the total records for each city, was recorded in Ourense with 41,395 spores/m^3^, followed by Vigo with 35,071 spores/m^3^, Santiago de Compostela with 19,148 spores/m^3^, and Lugo with 16,201 spores/m^3^. When calculating the annual averages of *Alternaria* spores, to normalize the data, Ourense retained first place with 2152 spores/m^3^, while Santiago de Compostela recorded the lowest concentrations (957 spores/m^3^), coinciding also with the lowest daily average (only three spores/day). Vigo and Lugo recorded intermediate annual averages (1948 and 1246 spores/m^3^, respectively), with daily averages below seven spores/day.

To obtain additional information on the representativeness of *Alternaria* spores in the air, the number of days of absence was calculated for each locality. The results showed that Santiago de Compostela and Lugo presented a more marked absence, with an average of 206 and 180 days per year, respectively, compared with the other two stations (Vigo recorded 154 days and Ourense 126 days) (Table 1). 

The days on which values higher than 100 spores/m^3^ were recorded during the whole study period invariably occurred in July, but they were scarce (eight in Ourense, seven in Santiago de Compostela, six in Vigo) or even null, as at the Lugo station. The maximum daily peak was recorded in all four localities in July, with the highest value in Santiago de Compostela (653 spores/m^3^; 9 July 1997) and the lowest value in Lugo (87 spores/m^3^; 26 July 2003). In Ourense and Vigo, they ranged between 332 spores/m^3^ on 27 July 1999, and 370 spores/m^3^ on 10 July 1997, respectively.

Santiago de Compostela, Vigo, and Lugo were the sites with the highest precipitation during the study period (between 1838 and 1013 mm), while Ourense was the driest (856 mm); relative humidity did not show notable variations (73–80%). Ourense recorded the highest maximum temperatures (21.8 °C), while at the other locations the maximum temperature was three degrees below this value. The lowest minimum temperature was recorded at Lugo (6.9 °C), while the highest was detected in Vigo (11.6 °C). The average temperature range varied between 12.3 °C in Lugo and 15.2 °C in Ourense.

In terms of the average concentrations of *Alternaria* spores throughout the year, the distribution shows a uniform pattern across the four locations. Spore concentrations were low in the first three and last three months of the year, coinciding with variations in the maximum, minimum, and average temperatures, which were optimal for their presence (Figure 2).

The MSS–G, calculated using the averages of the years at each location, started in May and ended in October for all stations (Table 1). The onset occurred within a few days of difference comparing the Ourense and Vigo stations (1 and 3 May, respectively), more than 10 days in advance of Santiago de Compostela and Lugo (14 and 13 May, respectively). The same occurred with the dates on which the MSS–G ended: while it took place in October at all stations, in Ourense and Vigo it was later (25 and 27 October, respectively) than in Santiago and Lugo (18 and 8 October, respectively), so that the duration of the MSS–G at the latter stations was shorter (158 and 149 days compared with 178 days at the other two locations).

During the MSS–G, some variations with respect to the climatic data of the global period were detected at all locations (Table 1). Vigo was the wettest city (480 mm) and Lugo the driest (220 mm), while relative humidity ranged from 67% in Ourense to 78% in Santiago. Ourense showed the highest average values of maximum and mean temperature (27.4 °C and 19.9 °C, respectively) and Lugo the lowest values of minimum temperature (11 °C) and mean temperature (17.2 °C).

### 3.2. Spore Concentration at Each Location

**Figure 3:**
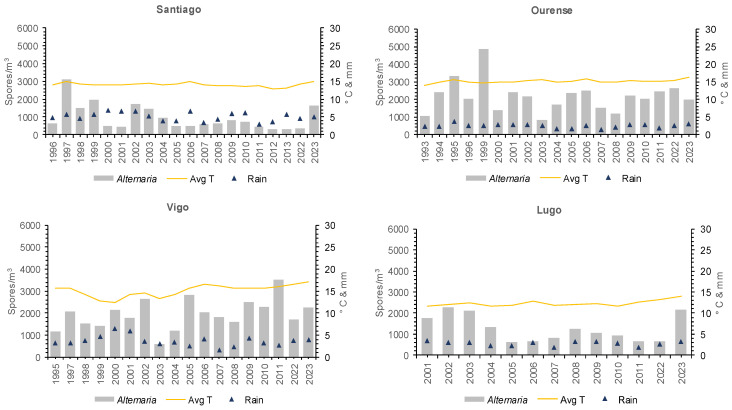
Total annual *Alternaria* spore concentrations, average temperature, and rainfall of each sampling location during the studied period in Santiago de Compostela, Ourense, Vigo, and Lugo.

*Alternaria* spore concentrations exhibit notable differences in each city from year to year (Figure 3, Table 2). In Santiago de Compostela, annual totals ranged from 3107 spores/m^3^ (1997) to 313 spores/m^3^ (2013), which represents the lowest annual record in the entire study area. In Ourense, 4847 spores/m^3^ were counted in 1999, representing the highest annual record of all stations, while in Vigo the annual maximum occurred in 2011 with 3520 spores/m^3^. In 2003, the lowest values coincided in Ourense (809 spores/m^3^) and Vigo (571 spores/m^3^). In Lugo, the highest concentrations were in 2002 (2285 spores/m^3^) and the lowest in 2022 (638 spores/m^3^).

The total annual spore concentration is related to the MSS–A concentrations in all years and locations (Table 2). The highest concentration during MSS–A was recorded in Ourense (4398 spores/m^3^ in 1999) and the lowest in Santiago (284 spores/m^3^ in 2013). 

In the years with the highest spore concentration during MSS–A, in Ourense and Vigo, precipitation and humidity were lower and temperatures higher than in the MSS–A with the lowest spore concentration. 

The earliest date for the start of the MSS–A was March 9 in Lugo in 2022, and the latest date for the end of the MSS–A was November 14 in Lugo (2002) and in Ourense (2003). The duration ranged from 124 days in the Santiago MSS–A (2013) to 244 days in Ourense (2003).

The monthly peaks were located in summer or autumn, depending on the year and location (Table S1). In Santiago, they occurred between June and September (mainly in July), with a maximum of 1941 spores/m^3^ in July 1997. In Ourense, they occurred between June and October (predominantly in August), although the month with the most spores was July 1999 with 2026 spores/m^3^. In Vigo, the monthly maximum occurred between July and October (mainly in August), and the highest value was 1049 spores/m^3^ in August 2011. In Lugo, the monthly maximum occurred between July and September (predominantly in August), with a peak of 643 spores/m^3^ in August 2003.

### 3.3. Relationship with Meteorological Parameters

**Figure 4:**
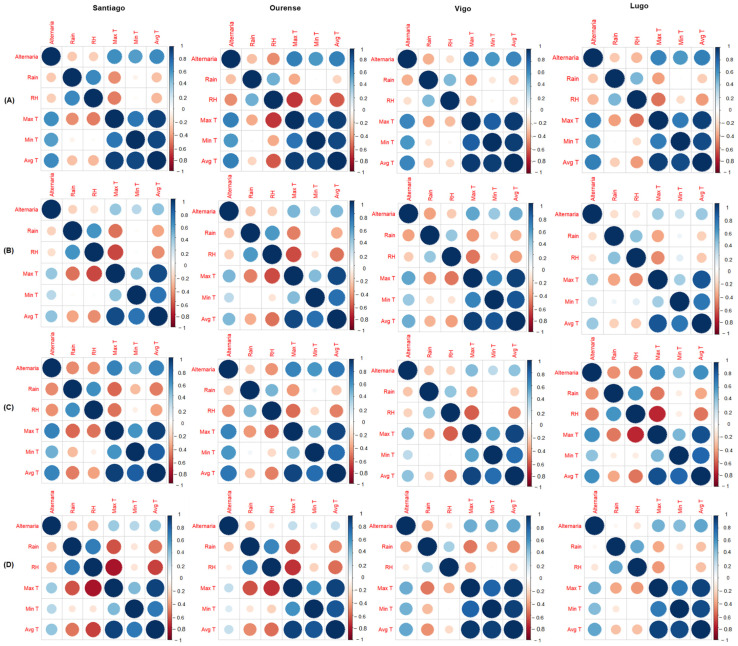
Correlograms between daily *Alternaria* concentrations in the study localities (**A**) Total period of study, (**B**) Main Spore Season–Global (MSS–G), (**C**) Main Spore Season–Annual (MSS–A) (maximum year), and (**D**) MSS–A (minimum year) and the meteorological variables, by Spearman’s correlation method. The meteorological variables were rainfall (Rain), relative humidity (RH), maximum temperature (Max T), minimum temperature (Min T), and mean temperature (Avg T). The size of the circles represents the strength of the association between variables. The blue color scale represents positive associations, and the red color scale represents negative associations; the intensity of the color represents the strength of the association.

The influence of meteorological variables on *Alternaria* concentrations during the entire study period and of the MSS, recorded in the air at each location, was statistically evaluated by means of a Spearman correlation test (Figure 4, Table S2). In all cities, the temperature (maximum, minimum, and average) had a high and positive correlation with the presence of spores, and rainfall and relative humidity had a negative influence, with lower rho values. Both variables had the same type of correlation during MSS–G and in MSS–A, where the highest concentrations were recorded in each city. However, in the MSS–A in which the lowest concentrations were recorded, rainfall was not correlated with the presence of *Alternaria* in Lugo, nor with relative humidity in Ourense, Vigo, or Lugo.

**Figure 5:**
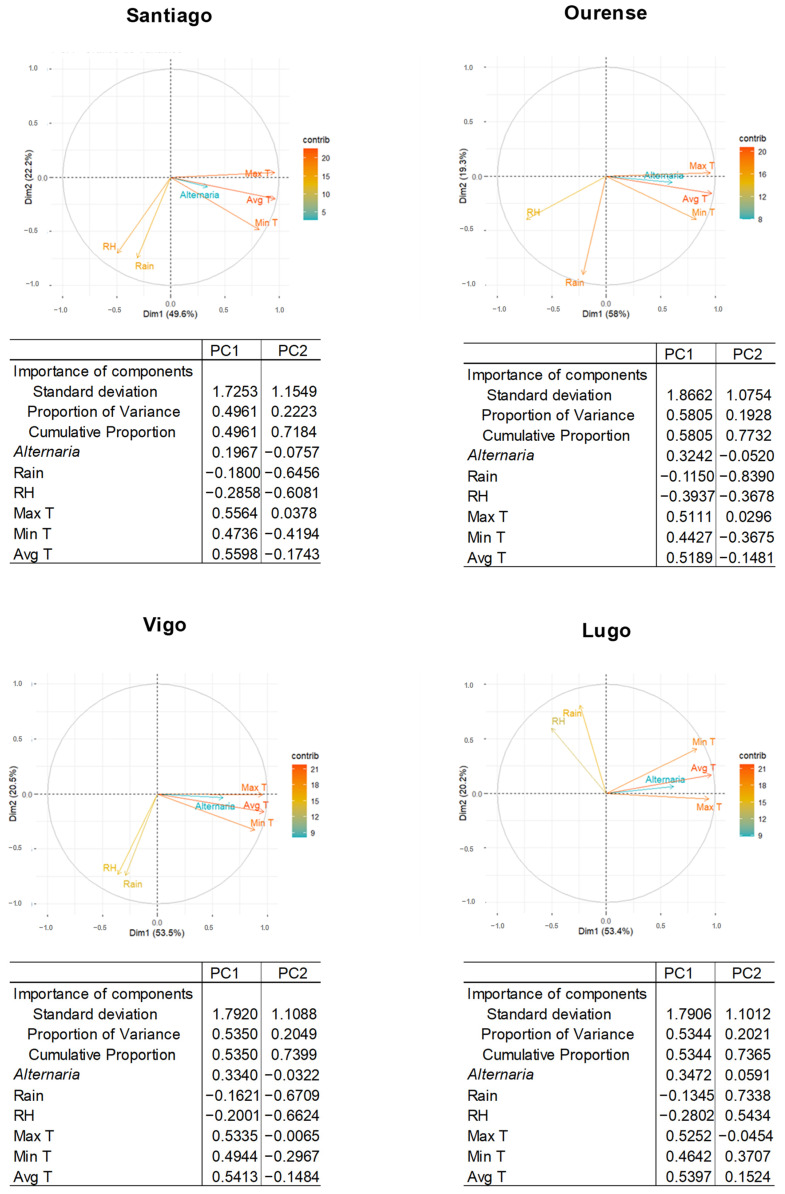
Principal component analysis in the study localities. Principal component one (PC1), principal component two (PC2). The meteorological variables were rainfall (Rain), relative humidity (RH), maximum temperature (Max T), minimum temperature (Min T), and average temperature (Avg T).

The PCA was performed taking into account the total of the study period because the behavior of the correlations of the total study period was the same as in the MSS–G (Table S2) and because the values were higher (Figure 5). This test showed that PC1 and PC2 accounted for more than 68% of the variance in the data. *Alternaria* concentrations and temperature (maximum, minimum, and average) had a greater contribution in PC1 in all cities. Rainfall and relative humidity contributed more in PC2, except relative humidity in Ourense, which contributed slightly more in PC1. It can therefore be stated that temperature is the meteorological variable that has the greatest influence on the concentrations of these spores in the air at all locations. The analysis of vector angles reaffirms the type of correlation obtained in Spearman’s test. In addition, *Alternaria* concentrations are more strongly correlated with temperature than with rainfall and relative humidity. In Santiago de Compostela, Ourense, and Lugo, this association is stronger with average temperature, while in Vigo it is stronger with maximum temperature.

**Figure 6:**
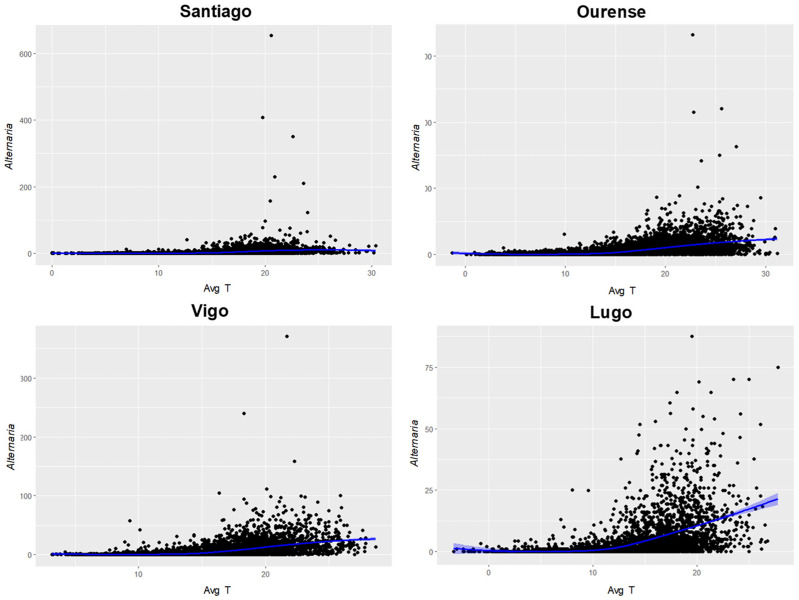
Relationship between daily *Alternaria* spore concentrations and mean daily temperature in Santiago de Compostela, Ourense, Vigo, and Lugo during the studied period. The blue line represents a LOESS regression, and the blue area represents the 95% confidence interval, α = 0.75.

By analyzing the relationship between the average daily temperature of each location and *Alternaria* concentrations using the LOESS regression models, it was evident that the average temperature value with the highest predicted airborne concentration of *Alternaria* in Santiago de Compostela was 25.5 °C, in Ourense 31.2 °C, in Vigo 28.7 °C, and in Lugo 27.8 °C. Concentrations tended to 0 spores/m^3^ when the average temperature fell below 11 °C in all cities (Figure 6 and Figure S1).

## 4. Discussion

### 4.1. Temporal Variation of Alternaria Atmospheric Concentrations

In the present study, focused on the northwest of the Iberian Peninsula, with a much larger number of years analyzed compared with other studies published in the same area [43,44,45,46], it is confirmed that *Alternaria* concentrations are lower in the cities of northern Spain than those of central and southern cities [47]. Annual spore counts varied in the years studied, as has been reported for other locations in Spain [48,49]. Recio et al. [49] report annual variations for airborne *Alternaria* spores that range between 9212 and 18,811 in Malaga (Spain).

The highest yearly total of *Alternaria* spores in the 70 annual datasets analyzed was recorded in Ourense, followed by Vigo, Santiago de Compostela, and Lugo. This higher frequency of spores in the city of Ourense is further reinforced by the fact that it was the city with the lowest number of days of absence and the highest number of days with concentrations above 100 spores/day. In addition, the aforementioned geographical distribution is observed on a smaller territorial scale since the southernmost locations of Ourense and Vigo had the highest annual and daily concentrations. 

In the study area, MSS–G spans a single season, starting in May and ending in October, which is similar to other sites located in northern and central Europe [35,50,51]. In these areas, summers are usually not warm enough to interrupt the favorable period, and optimal conditions for fungal growth and reproduction are more constant [8,52]. In southern areas, by contrast, it is common to observe two spore seasons, one at the end of spring and the other in autumn, due to the high summer temperatures [47,51,53]. 

Spore concentration was always low in the first and last months of the year in all locations, but monthly peaks were detected mainly in summer in Santiago de Compostela and Lugo and in late summer to autumn in Ourense and Vigo, where the MSS–G lengthens. Likewise, in other localities such as Madrid, the highest levels occur in June, while in some Portuguese locations they are delayed until September [47]. 

The duration of MSS–G in the present study ranged from 149 to 178 days, which was not significantly different from those of other Spanish localities such as Madrid, Mérida, and Seville [47]. In other European countries, such as Ireland, the MSS for *Alternaria* has been reported as occurring between early July and early September [54]. In Kraków, Poland, the season starts in June and lasts until mid-September [55]. 

In Derby, UK, Corden and Millington [56] found that the onset date of the *Alternaria* MSS had clearly advanced from June to early June over the course of 25 years. In the present study, however, no trend of advancing or delaying the onset, termination, or duration of the MSS was detected. 

### 4.2. Factors Related to Alternaria Concentrations in the Air

The number of spores at a location is related to multiple factors, and it is likely that not all of them affect the concentration of atmospheric spores at different sites in the same way. Several authors have shown that the spatiotemporal variation of airborne *Alternaria* spores is influenced by meteorological variables. Temperature, solar radiation, and wind speed generally influence their presence, while relative humidity and rainfall influence their absence [31,32,57]. 

In the present investigation, the detected concentrations of *Alternaria* at the sampled locations were positively related to temperature and negatively related to rainfall and relative humidity, as in previous studies [44,58]. Temperature was the parameter that had the greatest influence on the concentrations of these spores in the air. This is consistent with the timing of the MSS, since the highest concentrations coincide with the warmest months, while the lowest concentrations occur in the coldest months, reflecting a close relationship with the increase or decrease in temperature. In this regard, Picornell et al. [8] reported that at relatively cold locations such as Pamplona, Tudela, or Valladolid, the optimum temperature range for *Alternaria* spore production is normally only reached during the summer. In light of this, the regression model was constructed using the average temperature, from which it appears that the average temperature favoring the highest presence of *Alternaria* spores in this region ranges between 25.5 and 31.2 °C and that temperatures below 11 °C are not favorable. These values differ from those reported for other Spanish cities (18.9 °C–25.2 °C), being higher in the present study region [8]. This may be due to the fact that in the northwestern part of the Iberian Peninsula, rainfall is more abundant and humidity is higher, so the optimum temperature must be higher to favor the spores’ presence. With the increase in air temperature, the acceptation capacity of air increases for water steam and causes a decrease in relative humidity [59,60]. This decrease in relative humidity, as demonstrated by our statistical analyses (Spearman rank correlation and PCA), promotes an increase in the concentrations of *Alternaria* spores, which are considered “dry air spores” [49].

Climate change is causing alterations in the average values of meteorological variables at a global level and, as a consequence, has induced the adaptation of various fungal species to areas where no increase in their concentrations had formerly been detected [61]. In this study, it is evident that, comparing the last two sampling years, temperatures (maximum, minimum, and average) increased by approximately 1 °C, rainfall and relative humidity decreased, and that consequently *Alternaria* concentrations generally increased in 2023 compared with 2022. In addition, the onset dates of MSS–A in Santiago de Compostela, Ourense, and Lugo were delayed in 2023 with respect to 2022. This type of aerobiological study over the course of several years allows such variations to be monitored and appropriate measures to be taken.

In addition to meteorological variables, other factors may also explain the differences between spore concentrations at different locations. Amounts of atmospheric spores are also related to local inputs. Although the aerobiological stations discussed in this study are located in urban areas, the availability of substrate in the environment may favor fungal development and consequently spore production. 

The greater number of spores identified in Ourense may be related to its greater proximity to agricultural areas than the other sampling stations. In addition, the location of this city in a deep valley may limit the dispersion of spores to more distant areas. In fact, it was at this same aerobiological station that the highest levels of pollen grains of the entire northwestern peninsular were detected [62,63]. Apangu et al. [64] showed that one of the sources of spores in urban areas comprises local agricultural areas and that cereal crops and pastures are important sources of *Alternaria* emission [65]. In this sense, Skjøth et al. [34] demonstrated that there are emissions between 1.2 × 10^10^ and 6.7 × 10^10^ *Alternaria* spores ha^−1^ during wheat and barley harvesting periods, which influence the high concentrations detected in urban areas of Copenhagen. Likewise, in the UK and Poland, this increase in atmospheric concentrations has been associated with the population dynamics of some aphid species, an aspect to be taken into account in future studies [66]. In contrast, the Santiago de Compostela station is located in a large garden area of the university campus, and it is very likely that the local component influences the bioaerosol more than airborne spores carried over longer distances. Moreover, routine lawn mowing and other maintenance of ornamental flora may result in lower availability of substrate suitable for the development of these fungi.

### 4.3. Health Effects of Exposure to Alternaria Spores

The prevalence of respiratory allergic diseases due to sensitization to *Alternaria* allergens is estimated to be about 4.4% worldwide and 6.1% in Europe. The incidence varies depending on the extent of the study, the population analyzed, and the country. It is estimated that in Germany it affects 6–7% of the population, and in Greece 23.5% [52,67,68]. In Spain, the prevalence of sensitivity to *Alternaria* rises to as much as 20% in Madrid [69], although regional studies report much lower values, from 0.2% to 1.9% [16,70,71]. In Cordoba, *Alternaria* is reported to be the fungus that produces the highest number of positive allergic reactions, with 32% of patients sensitive to fungi being sensitive to *Alternaria* [72].

The percentage of sensitizations in Galicia is low, and this may be related to the low daily concentrations detected in the air, as reported in the present investigation. The concentration of 100 spores/m^3^ per day is considered the threshold at which allergies are triggered [73], and the authors did not find many days exceeding these values at the study locations during the 70 annual sampling records. The length of time in which this fungus with allergenic properties is present in the atmosphere could also represent a potential allergy risk for sensitized individuals [52]. In this regard, the shorter duration of MSS in the area currently under investigation may be considered a less detrimental aspect for the allergic population than in other latitudes.

## 5. Conclusions

The low concentration of *Alternaria* in the northwestern part of the Spanish peninsula with respect to other more southern locations and its relationship with temperature are confirmed at all the sites analyzed. In addition, there is only one season per annum when the spores emerge.

Taking into account the relationship between the concentration of *Alternaria* spores in the air and the development of allergic respiratory diseases, everything seems to indicate that there is no high potential risk for this bioallergen in the study area. However, given that the average temperature has been the meteorological parameter with the greatest influence on the concentration of spores and that this could rise with climate change, it is advisable to continue monitoring the air to verify the findings of this analysis over the long term.

## Figures and Tables

**Table 1:** Study period in each locality, *Alternaria* concentration values (total spores—spores/m^3^), annual average (spores/m^3^), daily average (spores/m^3^), average of days equal to 0, days greater than 100, maximum daily value (spores/m^3^), peak date of maximum daily value), and average values of meteorological variables (rainfall, relative humidity—RH; maximum temperature—Max T, minimum—Min T, and average—Avg T). Total (spores/m^3^) and annual mean (spores/m^3^) concentrations of *Alternaria* during Main Spore Season–Global (MSS–G), start and end date of MSS–G, duration, and average values of meteorological variables. The standard deviations of the average values are shown in parentheses.

	Santiago	Ourense	Vigo	Lugo
Study Period				
Years analyzed	20	19	18	13
	1996–2013; 2022–2023	1993–1996; 1999–2011; 2022–2023	1995; 1997–2011; 2022–2023	2001–2011; 2022–2023
Total spore count	19,148	41,395	35,071	16,201
Average annual	957 (723.59)	2152 (899.81)	1948 (688.71)	1246 (623.73)
Average daily	3 (12.32)	6 (11.95)	6 (12.17)	4 (7.60)
Average days equal to 0	206 (36.92)	126 (15.61)	154 (27.63)	180 (37.50)
Days over 100	7	8	6	0
Peak value	653	332	370	87
Peak date	9 July 1997	27 July 1999	10 July 1997	26 July 2003
Rainfall (mm)	1838 (431.65)	856 (197.02)	1352 (435.11)	1013 (195.71)
RH (%)	80 (11.34)	73 (11.58)	74 (13.18)	80 (9.75)
Max T (°C)	18.8 (5.94)	21.8 (7.50)	18.8 (5.18)	18.3 (6.79)
Min T (°C)	9.6 (4.54)	8.7 (5.43)	11.6 (4.21)	6.9 (5.33)
Avg T (°C)	14.1 (4.81)	15.2 (6.00)	15.1 (4.41)	12.3 (5.48)
Mean Spore Season–Global				
Total spore count	17,254	37,410	31,815	14,017
Average annual	863 (656.24)	1969 (793.22)	1767 (622.96)	1078 (574.70)
Start	14 May	1 May	3 May	13 May
End	18 October	25 October	27 October	8 October
Lenght (days)	158	178	178	149
Rainfall (mm)	437 (219.17)	316 (133.00)	480 (201.00)	220 (72.40)
RH (%)	78 (2.52)	67 (1.87)	74 (5.92)	76 (3.11)
Max T (°C)	23.4 (1.17)	27.4 (2.04)	22.1 (2.52)	24.0 (1.74)
Min T (°C)	13.2 (1.02)	12.6 (1.48)	14.5 (2.13)	11.0 (1.19)
Avg T (°C)	18.2 (1.06)	19.9 (1.65)	18.3 (2.17)	17.2 (1.48)

**Table 2:** Maximum (max) and minimum (min) annual total *Alternaria* spores at each location (spores/m^3^). Range of dates and spore concentration (spores/m^3^) in the Main Spore Season–Annual (MSS–A) and average values of meteorological variables (rainfall, relative humidity—RH, maximum temperature—Max T, minimum—Min T, and mean—Avg T) for that period.

					Rainfall	RH	Max T	Min T	Avg T
Sampling Station	Year	Total	MSS–A	Spores/m^3^	(mm)	(%)	(°C)	(°C)	(°C)
Santiago	1997 (max)	3107	2 May–7 October	2822	664	81	23.03	13.05	18.04
	2013 (min)	313	6 June–7 October	284	257	78	24.69	14.09	18.68
Ourense	1999 (max)	4847	26 May–28 October	4398	293	66	28.45	13.88	21.16
	2003 (min)	809	16 March–14 November	734	480	67	25.86	11.8	18.83
Vigo	2011 (max)	3520	17 May–28 October	3175	294	79	24.85	17.55	20.79
	2003 (min	571	19 Mar–14 November	522	586	69	17.06	11.10	14.10
Lugo	2002 (max)	2285	4 May–2 October	2070	209	73	22.13	10.20	15.53
	2005 (min)	638	18 April–22 September	575	249	76	23.08	10.02	16.63

## Data Availability

The datasets generated and/or analyzed during the current study are available from the corresponding author on reasonable request.

## Supplementary Materials

The following supporting information can be downloaded at: https://www.mdpi.com/article/10.3390/jof10100681/s1, Figure S1: Residual plot from LOESS regression fitted to *Alternaria* concentration data and average daily temperature in each study city. The solid line is a LOESS curve, fitted to the residuals with α = 0.75; Table S1: Maximum month of concentration (spores/m^3^) of *Alternaria* in each year of study in Santiago de Compostela, Ourense, Vigo, and Lugo; Table S2: Spearman’s correlation analysis between *Alternaria* concentration (total period of study, MSS–G (Main Spore Season Global), MSS–A (Annual Main Spore Season Global) (maximum and minimum year)), and meteorological variables (rainfall (mm), relative humidity (RH—%), maximum, minimum, and average temperatures (Max T, Min T, Avg T—°C)); No statistical significance in bold (*p* > 0.05).
