# Supplementary material for: Airborne Alternaria Spores: 70 Annual Records in Northwestern Spain
Source: J Fungi (Basel). 2024 Sep 29;10(10):681. doi: 10.3390/jof10100681 (PMC11508870; doi:10.3390/jof10100681)
Supplement: Supplementary file 1 [file jof-10-00681-s001.zip › jof-3204754-supplementary.pdf]

Table S1. Maximum month of concentration (spores/m³) of *Alternaria* in each year of study in Santiago de Compostela, Ourense, Vigo and Lugo.

Santiago			Ourense			Vigo			Lugo		
**Month**	**Year**	**Spores/m³**	**Month**	**Year**	**Spores/m³**	**Month**	**Year**	**Spores/m³**	**Month**	**Year**	**Spores/m³**
August	**1996**	221	September	**1993**	200	October	**1995**	334	August	**2001**	551
July	**1997**	1941	August	**1994**	811	July	**1997**	823	July	**2002**	607
August	**1998**	336	July	**1995**	897	September	**1998**	268	August	**2003**	643
July	**1999**	1013	August	**1996**	653	July	**1999**	969	July	**2004**	475
July	**2000**	197	July	**1999**	2026	August	**2000**	674	September	**2005**	186
July	**2001**	106	August	**2000**	366	August	**2001**	658	August	**2006**	172
July	**2002**	407	August	**2001**	741	August	**2002**	798	August	**2007**	267
August	**2003**	367	September	**2002**	642	August	**2003**	144	August	**2008**	455
July	**2004**	291	October	**2003**	187	September	**2004**	276	July	**2009**	291
September	**2005**	234	July	**2004**	452	August	**2005**	961	July	**2010**	251
August	**2006**	130	August	**2005**	756	July	**2006**	367	August	**2011**	134
August	**2007**	162	August	**2006**	598	September	**2007**	404	July	**2022**	179
September	**2008**	186	August	**2007**	410	September	**2008**	380	August	**2023**	631
September	**2009**	219	September	**2008**	443	September	**2009**	756			
July	**2010**	170	August	**2009**	461	September	**2010**	673			
August	**2011**	119	September	**2010**	542	August	**2011**	1049			
August	**2012**	106	August	**2011**	676	September	**2022**	706			
August	**2013**	108	September	**2022**	677	August	**2023**	525			
July	**2022**	105	June	**2023**	553						
July	**2023**	429									

Table S2. Spearman's correlation analysis between *Alternaria* concentration (total period of study, MSS−G (Main Spore Season Global), MSS-A (Annual Main Spore Season Global) (maximum and minimum year)), and meteorological variables (rainfall (mm), relative humidity (RH −%), maximum, minimum and average temperatures (Max T, Min T, Avg T −°C)); No statistical significance in bold ($p > 0.05$).

Sampling Station	Total Period of Study				
	Rain	RH	Max T	Min T	Avg T
Santiago	−0.26414	−0.24935	0.61736	0.55584	0.63639
Ourense	−0.29544	−0.46151	0.68810	0.57921	0.68832
Vigo	−0.35865	−0.17176	0.65733	0.58782	0.65335
Lugo	−0.26533	−0.32429	0.66965	0.57992	0.68477
**MSS-G**					
Santiago	−0.22418	−0.17794	0.38387	0.27326	0.40101
Ourense	−0.28556	−0.24628	0.45802	0.26919	0.44179
Vigo	−0.40679	−0.25791	0.52044	0.37650	0.49023
Lugo	−0.17306	−0.14601	0.35764	0.23753	0.37838
**MSS-A (maximum year)**					
Santiago	−0.47785	−0.45563	0.67928	0.48181	0.66863
Ourense	−0.25716	−0.44573	0.66547	0.58817	0.72500
Vigo	−0.27086	−0.22360	0.43273	0.26749	0.40264
Lugo	−0.45215	−0.49174	0.61835	0.40844	0.61310
**MSS−A (minimum year)**					
Santiago	−0.28788	−0.32400	0.36225	0.28968	0.39379
Ourense	−0.26659	**−0.09360**	0.24539	0.14005	0.23777
Vigo	−0.35891	**−0.11591**	0.49674	0.47625	0.51438
Lugo	**−0.04351**	**−0.13978**	0.47459	0.43502	0.52086

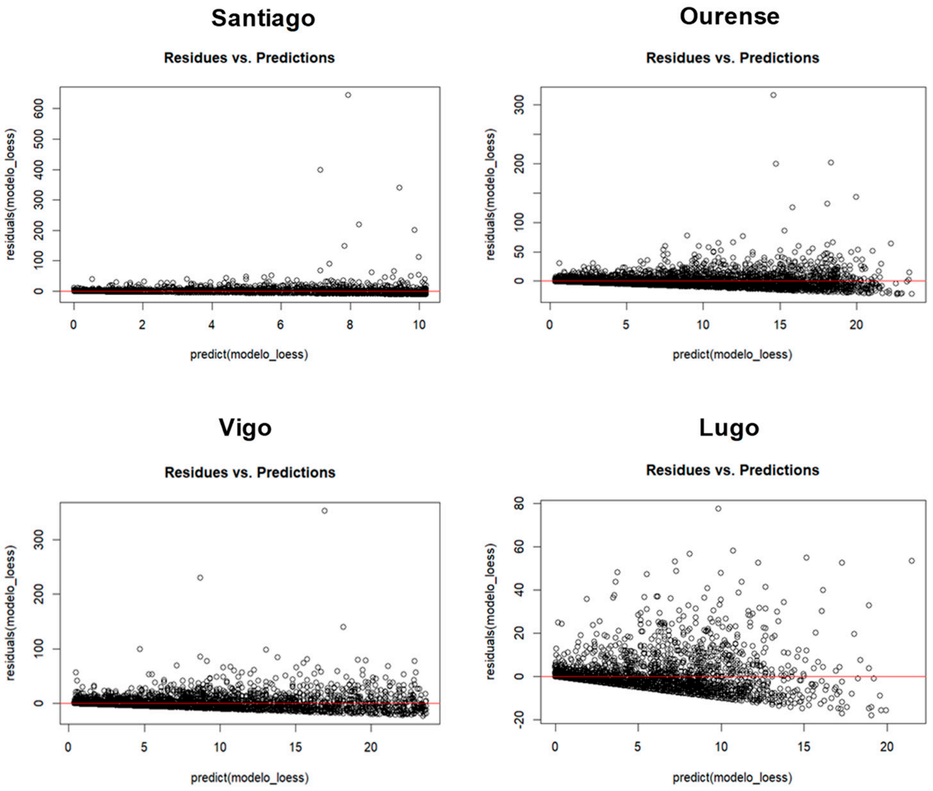

Figure S1. Residual plot from LOESS regression fitted to *Alternaria* concentration data and average daily temperature in each study city. The solid line is a LOESS curve, fitted to the residuals with $\alpha = 0.75$
